# Canakinumab as first-line biological therapy in Still’s disease and differences between the systemic and the chronic-articular courses: Real-life experience from the international AIDA registry

**DOI:** 10.3389/fmed.2022.1071732

**Published:** 2022-12-22

**Authors:** Antonio Vitale, Valeria Caggiano, Maria Cristina Maggio, Giuseppe Lopalco, Giacomo Emmi, Jurgen Sota, Francesco La Torre, Piero Ruscitti, Elena Bartoloni, Giovanni Conti, Claudia Fabiani, Irene Mattioli, Carla Gaggiano, Fabio Cardinale, Lorenzo Dagna, Corrado Campochiaro, Roberto Giacomelli, Alberto Balistreri, Katerina Laskari, Abdurrahman Tufan, Gaafar Ragab, Ibrahim A. Almaghlouth, Ewa Więsik-Szewczyk, Rosa Maria Pereira, Bruno Frediani, Florenzo Iannone, Petros P. Sfikakis, Luca Cantarini

**Affiliations:** ^1^Department of Medical Sciences, Surgery and Neurosciences, Research Center of Systemic Autoinflammatory Diseases and Behçet’s Disease Clinic, University of Siena, Siena, Italy; ^2^University Department Pro.Sa.M.I. “G. D’Alessandro”, University of Palermo, Palermo, Italy; ^3^Rheumatology Unit, Department of Emergency and Organ Transplantation, University of Bari Aldo Moro, Bari, Italy; ^4^Department of Experimental and Clinical Medicine, University of Florence, Florence, Italy; ^5^Centre for Inflammatory Diseases, Monash Medical Centre, Monash University Department of Medicine, Melbourne, VIC, Australia; ^6^Pediatric Rheumatology Center, Department of Pediatrics, Ospedale “Giovanni XXIII”, Azienda Ospedaliero Universitaria Consorziale Policlinico, Bari, Italy; ^7^Rheumatology Unit, Department of Biotechnological and Applied Clinical Sciences, University of L’Aquila, L’Aquila, Italy; ^8^Rheumatology Unit, Department of Medicine, University of Perugia, Perugia, Italy; ^9^Pediatric Nephrology and Rheumatology Unit, Azienda Ospedaliera Universitaria (AOU), “G. Martino” Messina, Italy; ^10^Ophthalmology Unit, Department of Medicine, Surgery and Neurosciences, University of Siena, Siena, Italy; ^11^Division of Immunology, Transplants and Infectious Diseases, Università Vita-Salute San Raffaele, Milan, Italy; ^12^Unit of Immunology, Rheumatology, Allergy and Rare Diseases, IRCCS Ospedale San Raffaele, Milan, Italy; ^13^Rheumatology, Immunology and Clinical Medicine Unit, Department of Medicine, Università Campus Bio-Medico di Roma, Selcetta, Italy; ^14^Joint Academic Rheumatology Program, The First Department of Propaedeutic and Internal Medicine, School of Medicine, National and Kapodistrian University of Athens, Athens, Greece; ^15^Division of Rheumatology, Department of Internal Medicine, Gazi University Faculty of Medicine, Ankara, Turkey; ^16^Rheumatology and Clinical Immunology Unit, Department of Internal Medicine, Faculty of Medicine, Cairo University, Giza, Egypt; ^17^Faculty of Medicine, New Giza University, Giza, Egypt; ^18^Rheumatology Unit, Department of Medicine, College of Medicine, King Saud University, Riyadh, Saudi Arabia; ^19^College of Medicine Research Center, College of Medicine, King Saud University, Riyadh, Saudi Arabia; ^20^Department of Internal Medicine, Pulmonology, Allergy and Clinical Immunology, Central Clinical Hospital of the Ministry of National Defence, Military Institute of Medicine, Warsaw, Poland; ^21^Rheumatology Division, Hospital das Clínicas da Faculdade de Medicina da Universidade de São Paulo (HCFMUSP), São Paulo, Brazil

**Keywords:** AOSD, adult onset Still’s disease, sJIA, systemic juvenile idiopathic arthritis, autoinflammatory diseases, biological therapy, interleukin-1

## Abstract

**Objective:**

Interleukin (IL)-1 inhibitors are largely employed in patients with Still’s disease; in cases with refractory arthritis, IL-6 inhibitors have shown to be effective on articular inflammatory involvement. The aim of the present study is to assess any difference in the effectiveness of the IL-1β antagonist canakinumab prescribed as first-line biologic agent between the systemic and the chronic-articular Still’s disease.

**Methods:**

Data were drawn from the retrospective phase of the AutoInflammatory Disease Alliance (AIDA) international registry dedicated to Still’s disease. Patients with Still’s disease classified according to internationally accepted criteria (Yamaguchi criteria and/or Fautrel criteria) and treated with canakinumab as first-line biologic agent were enrolled.

**Results:**

A total of 26 patients (17 females, 9 males; 18 patients developing Still’s disease after the age of 16 years) were enrolled; 16 (61.5%) patients suffered from the systemic pattern of the disease; 10 (38.5%) patients suffered from the chronic-articular type. No differences were observed between the systemic and the chronic-articular Still’s disease in the frequency of complete response, of flares after the start of canakinumab (*p* = 0.701) and in the persistence in therapy (*p* = 0.62). No statistical differences were observed between the two groups after 3 months, 12 months and at the last assessment in the decrease of: the systemic activity score (*p* = 0.06, *p* = 0.17, *p* = 0.17, respectively); the disease activity score on 28 joints (*p* = 0.54, *p* = 0.77, *p* = 0.98, respectively); the glucocorticoid dosage (*p* = 0.15, *p* = 0.50, and *p* = 0.50, respectively); the use of concomitant disease modifying anti-rheumatic drugs (*p* = 0.10, *p* = 1.00, and *p* = 1.00, respectively). No statistically significant differences were observed in the decrease of erythrocyte sedimentation rate (*p* = 0.34), C reactive protein (*p* = 0.48), and serum ferritin levels (*p* = 0.34) after the start of canakinumab.

**Conclusion:**

Canakinumab used for Still’s disease has been effective in controlling both clinical and laboratory manifestations disregarding the type of disease course when used as first-line biotechnological agent. These excellent results might have been further enhanced by the early start of IL-1 inhibition.

## Introduction

Still’s disease is a systemic polygenic autoinflammatory condition mainly characterized by fever, maculopapular skin rash, arthritis, arthralgia, serositis, and hepato-splenomegaly. Still’s disease may be a life-threatening condition when patients develop the macrophage activation syndrome or other severe affections including pulmonary arterial hypertension, lung fibrosis, and disseminated intravascular coagulation. In patients with no concomitant macrophage activation syndrome, erythrocyte sedimentation rate (ESR), C reactive protein (CRP), and ferritin serum levels are generally increased during disease activity (1).

In the past decades, Still’s disease had been distinguished into systemic juvenile idiopathic arthritis (sJIA) for patients experiencing disease onset before the age of 16 and adult-onset Still’s disease (AOSD) for patients with a later disease onset. However, based on similar pathogenesis, overlapping clinical manifestations and organ involvement, sJIA and AOSD are currently considered to represent a disease continuum of the same clinical entity arising in different ages (2, 3).

According to the clinical course, Still’s disease may be distinguished into a “systemic” type and a “chronic-articular” type. The former includes patients mainly suffering from daily spiking fevers and systemic inflammation with skin, serosal and lymph node involvement; the latter includes patients with a prominent articular affection and less pronounced systemic inflammatory features. Still’s disease can be distinguished into a monocyclic and polycyclic type, but this distinction has no relevance with respect to a prognostic stratification (4).

Diagnosis of Still’s disease is primarily clinical and requires the exclusion of infections, neoplasms, autoimmune disorders and other autoinflammatory diseases. Different sets of criteria are currently available for diagnostic and classification purposes, with Yamaguchi’s criteria and Fautrel’s criteria being the most frequently employed in adults and the International League of Associations for Rheumatology (ILAR) criteria and/or the Pediatric Rheumatology INternational Trials Organization (PRINTO) criteria used in the pediatric setting (5–8).

Waiting for new clinimetric tools to measure disease severity and activity (9), the systemic Pouchot’s score modified by Rau et al. at current (10); this is also useful in identifying patients at higher risk of death (11). The articular involvement may be assessed using the disease activity score based on 28 joints (DAS28) in adult patients or the juvenile arthritis disease activity score based on 27 joints (JADAS27) in pediatric patients (12, 13).

Treatment with biotechnological anti-interleukin (IL)-1 agents is recommended in patients with active disease especially in cases refractory to glucocorticoids and conventional disease modifying anti-rheumatic drugs (cDMARDs), avoiding a long-term glucocorticoids exposure (14–18). Agents blocking IL-6 are also recommended and may represent a valuable option in patients refractory to other treatment choices, as for joint involvement in Still’s disease (14–16). Randomized control trials and real-world studies describe the efficacy of anti-IL-1 inhibition on the articular inflammatory involvement, with a significant decrease in the number of tender joints, swollen joints, DAS28 and JADAS27 (19–21). On the other hand, other evidence suggests that articular involvement responds to IL-1 inhibitors less quickly, especially in patients with a longer time between disease onset and the start of anti-IL-1 agents (21, 22).

With this background, the present study was performed to assess any difference in the effectiveness of canakinumab prescribed as first-line biologic agent between the systemic and the chronic-articular Still’s disease.

## Materials and methods

This study has been performed based on data collected in the retrospective phase of the international Registry on Still’s disease promoted by the AutoInflammatory Disease Alliance (AIDA) Network (23).

The objective of this paper is to investigate the role of the anti-IL-1β canakinumab administered in patients with Still’s disease as first-line biologic agent, looking for any difference in the therapeutic outcome between patients with the systemic type and patients with the chronic-articular type, disregarding age at disease onset.

The endpoints of the study are represented by the lack of statistically significant differences between patients with the systemic type and those with the chronic-articular type in terms of: frequency of complete response, frequency of partial response, frequency of flares during treatment, decrease in the systemic Pouchot and Rau score, DAS28, number of tender joints, number of swollen joints, physician global assessment (PhGA) of arthritis, patient’s global assessment (PGA) of arthritis, glucocorticoid sparing, cDMARDs sparing, decrease in laboratory markers ESR, CRP, and serum ferritin levels. Clinical outcomes were assessed at the 3-, 6-, and 12-month visits and at the last follow-up evaluation while on treatment with canakinumab. Laboratory outcomes were analyzed at the baseline and at the 3-month assessment.

**FIGURE 1:**
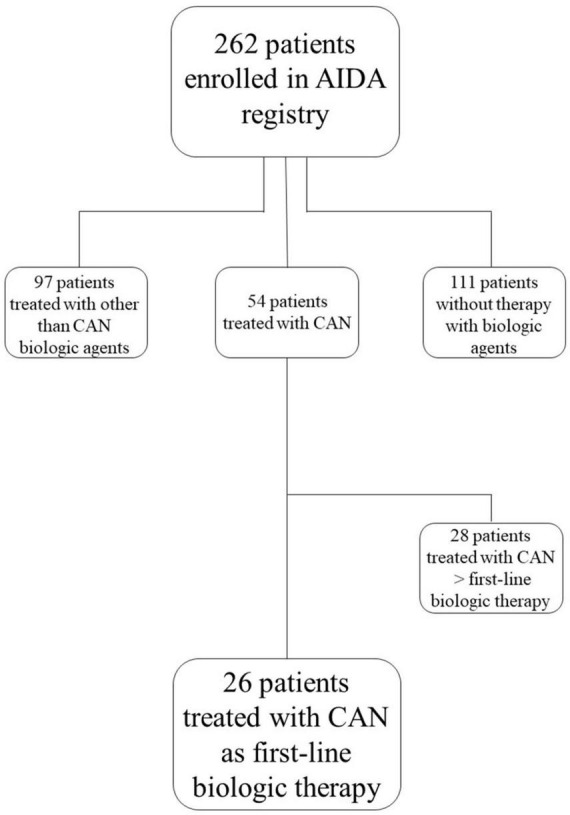
Study flow diagram illustrating the process leading to the selection of patients included in the present study among all patients recruited in the international AutoInflammatory Disease Alliance (AIDA) Registry dedicated to Still’s disease. AIDA, AutoInflammatory Disease Alliance; CAM, canakinumab.

Patients were retrospectively collected from the AIDA Registry according to the following features required by this study: diagnosis of Still’s disease classified according to Yamaguchi and/or Fautrel criteria (5, 6) in adult patients and to the ILAR criteria and/or the PRINTO criteria in patients aged less than 16 years (7, 8); signed consent/assent to participate to the AIDA Registry and studies; treatment with canakinumab as first line biologic agent. Figure 1 corresponds to the study flow diagram explaining the selection of the patients enrolled in the present study among all patients recruited in the AIDA Registry dedicated to Still’s disease. Patients were followed in 12 rheumatologic, immunologic or pediatric Centers joining the AIDA Network.

Complete response was defined as the complete resolution of all disease-related clinical manifestations with decrease of all laboratory inflammatory parameters within normal values. Partial response was defined as persistence of clinical manifestations with remarkable decrease in their severity and inflammatory laboratory parameters normalized or only slightly increased. Poor response included patients not meeting the definitions of complete and partial response. A relapse was defined as reappearance of Still’s disease related clinical manifestations leading to treatment change, the addition of a cDMARD or the increase of glucocorticoid dosages.

DAS28 values were calculated based on the CRP values. Glucocorticoids dosages were expressed as prednisone or equivalent (mg/day).

The modified Pouchot score was calculated on both pediatric-onset and adult-onset patients, based on the retrospective evaluation of disease manifestations recorded at the start of canakinumab and at the following time-points. Conversely, DAS28 was only calculated in adult patients, as articular involvement in pediatric patients is assessed with the JADAS27 score in the clinical practice.

The study has been approved by the Ethics Committee of Azienda Ospedaliero Universitaria Senese, Siena, Italy (AIDA Project; Ref. N. 14951) as part of the AIDA Program. The study protocol conformed to the tenets of the Declaration of Helsinki; informed consent was obtained from all patients at the time of the recruitment into the AIDA Registry for Still’s disease.

Descriptive statistics included mean, standard deviation (SD), median and interquartile range (IQR) values according to the data distribution at the Shapiro–Wilk test. For qualitative data, comparisons were performed using 4 × 2, 3 × 2, and 2 × 2 contingency tables applying Fisher exact test with Freeman-Halton extension. For quantitative data, Kruskal–Wallis test of ANOVA test were used for global assessments, whilst Student *t*-test or Mann-Whitney *U* test were used for pairwise comparisons, as required. Significance level was set at 95% (*p*-value < 0.05); all tests performed were two-sided. The SPSS software, version 24, was used for statistical computations.

## Results

Twenty-six patients (17 females, 9 males) treated with canakinumab as first-line biologic agent were enrolled. The mean age at disease onset was 31.88 ± 17.66 years; the mean age at diagnosis was 32.68 ± 17.61 years.

The 18 patients developing Still’s disease after the age of 16 years fulfilled Yamaguchi criteria; 14/18 (77.8%) of these patients fulfilled also Fautrel criteria. The ILAR criteria and PRINTO provisional criteria were fulfilled in 6/8 (75%) and 7/8 (87.5%) pediatric cases, respectively. Canakinumab was started in patients aged less than 16 years in 3 cases.

Sixteen (61.5%) patients suffered from the systemic pattern of disease; 10 (38.5%) patients suffered from the chronic-articular type. The median time from disease onset at the start of canakinumab was 12.5 (IQR = 43.25) months, 9.5 (IQR = 23.0) months among patients with chronic-articular pattern and 30 (IQR = 56) months among patients with the systemic pattern (*p* = 0.17).

Table 1 shows treatment approaches attempted prior to canakinumab administration and those combined with canakinumab at the start of the treatment.

**TABLE 1:** Summary of treatment approaches performed in the study group prior to and concomitantly with the start of canakinumab.

Treatments preceding canakinumab	Systemic group (16 patients)	Chronic-articular group (10 patients)	*p*-value
NSAIDs alone	8 (50%)	7 (70%)	0.325
Systemic glucocorticoids	13 (81.3%)	6 (60%)	0.422
cDMARDs	10 (62.5%)	3 (30%)	0.114
Methotrexate	8 (50%)	2 (20%)	0.134
Colchicine	2 (12.5%)	0 (0%)	0.254
Hydroxychloroquine	1 (6.25%)	1 (10%)	1.000
Cyclosporine	1 (6.25%)	0 (0%)	0.429
Sulfasalazine	1 (6.25%)	0 (0%)	0.429
**Treatments at the start of canakinumab**			
cDMARDs	9 (56.25%)	5 (50%)	0.760
Methotrexate 15 mg/week	4 (25%)	1 (10%)	0.355
Methotrexate 12.5 mg/week	0 (0%)	1 (10%)	0.206
Methotrexate 7.5 mg/week	1 (6.25%)	1 (10%)	1.000
Hydroxychloroquine 400 mg/day	1 (6.25%)	1 (10%)	1.000
Colchicine 1 mg/day	2 (12.5)	0 (0.0)	0.254
Cyclosporine 200 mg/day	1 (6.25%)	0 (0%)	0.429
Mesalazine 2,400 mg/day	0 (0%)	1 (10%)	0.206

cDMARDs, conventional disease modifying anti-rheumatic drugs; NSAIDS, non-steroidal anti-inflammatory drugs.

The following schedules of canakinumab administration were employed: 150 mg every 4 weeks in 8 (31%) patients; 300 mg every 4 weeks in 9 (34.6%) patients; 240 mg every 4 weeks in one (4%) patient; 4 mg/Kg/4 weeks in the 8 (31%) pediatric patients. In 5 (19.2%) patients the schedule was changed over time: in 2 cases the posology was increased from 150 mg/4 weeks to 300 mg every/4 weeks and from 150 mg/8 weeks to 150 mg/4 weeks because of inadequate response to the previous posology; 3 patients underwent a decrease in the frequency of administrations from 150 mg/4 weeks to 150 mg/5 weeks, from 4 mg/Kg every 4 weeks to 4 mg/Kg every 7 weeks, and from 240 mg/4 week to 240 mg every 4 months after a long-lasting disease remission. No statistically significant differences existed between groups (*p* = 0.10).

**FIGURE 2:**
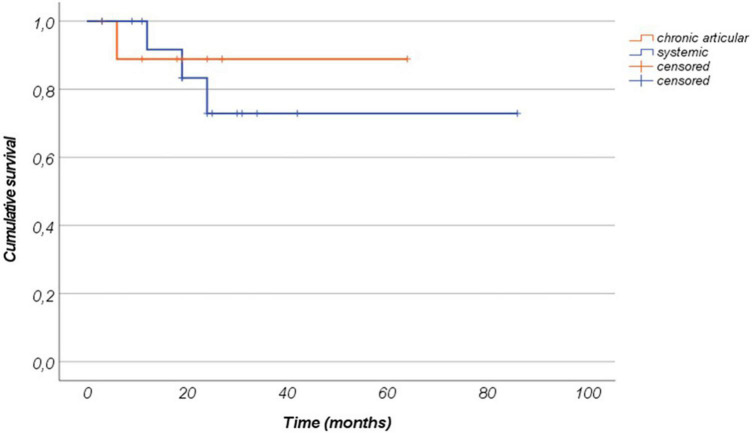
Canakinumab retention represented as a Kaplan–Meier plot among patients with the systemic form of Still’s disease and patients with the chronic-articular type. Time 0” corresponds to the start of canakinumab and the “event” corresponds to the treatment discontinuation.

**FIGURE 3:**
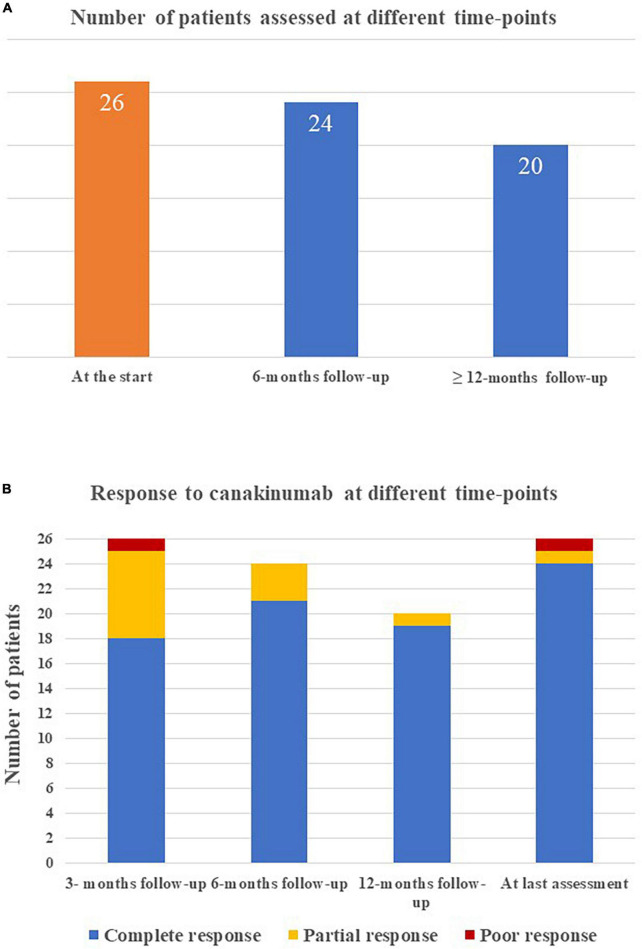
Graphical representation of **(A)** the number of patients reaching the different time-points during follow-up while on canakinumab treatment; **(B)** the frequency of complete response, partial response, and poor response in the whole group of patients enrolled at the different tume-points.

The mean duration of canakinumab treatment was 24.54 ± 17.91 months (range 3–86 months), with no differences between the systemic and the chronic-articular forms of the disease (25.75 ± 18.9 versus 22.6 ± 17.0 months, respectively, *p* = 0.62); Figure 2 graphically represents the persistence in canakinumab treatment of the two patients’ groups as a Kaplan–Meier plot. Twenty-four out of the total 26 patients (92.3%) had a minimum follow-up time of 6 months, and 20 (77%) of at least 12 months, as also reported in Figure 3A. The chronic-articular disease pattern affected 3 out of the 6 patients not reaching a 12-month follow-up period.

### Treatment effectiveness

Complete response was observed in 18/26 (69.2%) cases at 3-month assessment, 21/24 (87.5%) cases at 6-month assessment, 19/20 cases at 12-month evaluation and 25/26 (92.3%) cases at the last assessment.

As a whole, 16 disease flares were observed in 9 patients during the follow-up period (638 months of observation), corresponding to 0.012 flares/patient/year; 6/9 patients with flares were characterized by a systemic disease course and 3/9 patients showed a chronic disease course, with no differences between groups (*p* = 0.701).

Treatment discontinuation was observed in 4 (12.5%) patients due to long-term remission (2 patients with the systemic Still’s disease), in one patient with the chronic-articular type due to lack of efficacy and in 1 case owing to a scheduled pregnancy. Figure 3B shows the distribution of patients with no complete response according to the clinical course.

**FIGURE 4:**
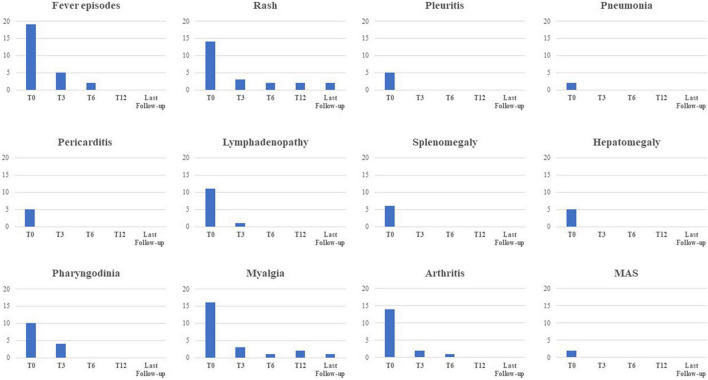
Frequency of the main clinical manifestations related to Still’s disease at the start of canakinumab and at the following time-points. MAS, macrophage activation syndrome.

Figure 4 shows the frequency of clinical manifestations at the start of canakinumab and at the 3-, 6-, and 12 month and last visit.

### Clinimetric changes during treatment

**FIGURE 5:**
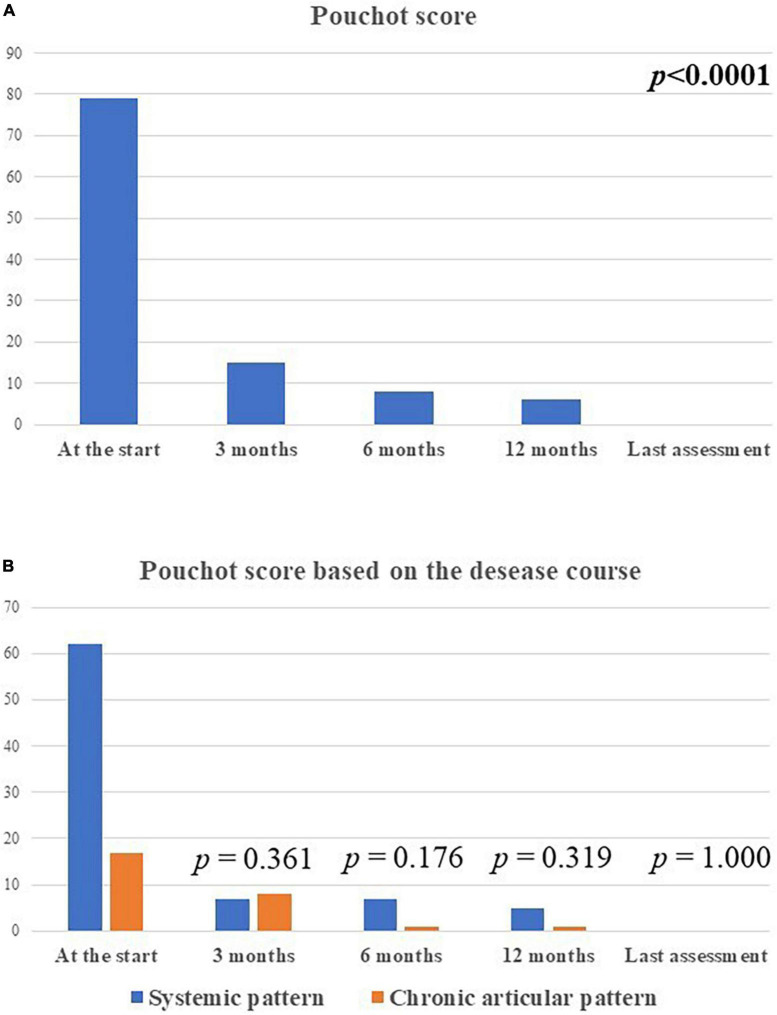
The total amount of the modified Pouchot score in the whole group of patients enrolled **(A)** and according with the disease course **(B)** at the different time-points. The total amount of the systemic modified Pouchot score was higher among patients with the systemic Still’s disease in relation with the higher systemic involvement in this patients compared to the chronic-articular type.

The median Pouchot score was 3.0 (IQR = 4.0) at the start of the treatment, 0.0 (IQR = 1) at the 3-month assessment, 0.0 (IQR = 1) at the 6-month assessment, and 0.0 (IQR = 0.0) at the last visit (*p* < 0.0001). No statistical differences were observed in the decrease of Pouchot score at the 3-month visit, the 12 month-visit and at the last assessment according with the disease course (*p* = 0.06, *p* = 0.17, *p* = 0.17, respectively). Figure 5 describes the amount of the Pouchot score at the different time-points.

**FIGURE 6:**
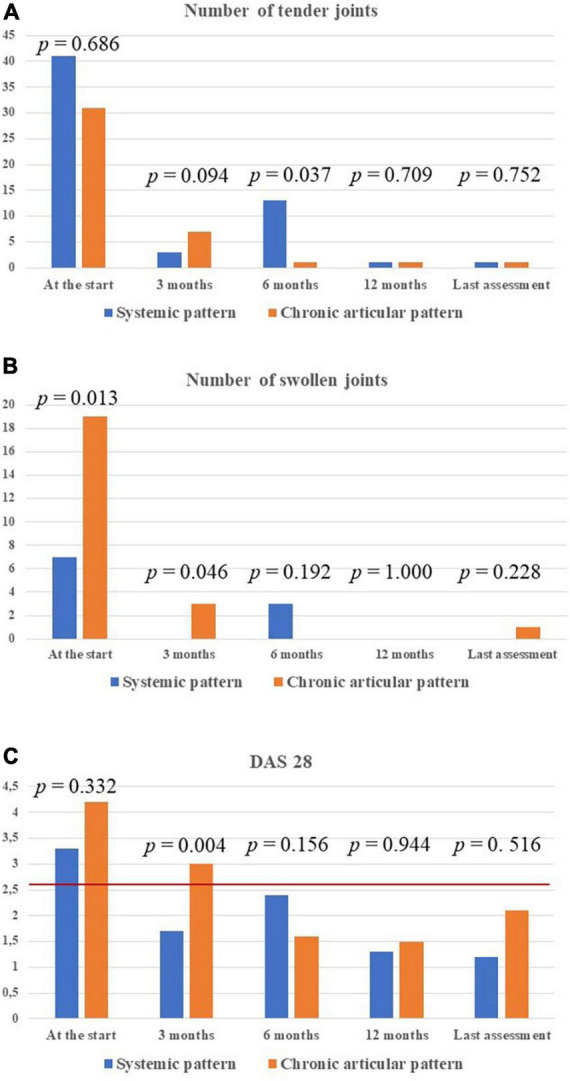
Detailed information about the treatment outcome relating to joints involvement among the 14 patients with arthritis. In particular, the total number of tender joints **(A)** and swollen joints **(B)** in all patients enrolled, distinguished according with Still’s disease course, has been provided. The DAS28 values have also been detailed in the two types of Still’s disease **(C)**. *P*-values refer to the differences between the two subgroups of patients in the number of tender joints, swollen joints and DAS28 values at each time-point. The red horizontal line in panel **(C)** indicates the DAS28 threshold below which arthritis may be considered in remission. DAS28, disease activity score based on 28 joints.

Arthritis was described in 14 (53.8%) patients, 5 with the chronic-articular course and 9 with the systemic course (*p* = 0.76). Two patients were affected by monoarthritis, 8 patients with oligoarthritis, and 4 patients with polyarthritis, with no statistically significant difference according to disease course (*p* = 0.56). Figures 6A, B provide details about the total number of tender and swollen joints recorded at the different time points while on canakinumab treatment. No statistically significant differences were observed in the number of tender and swollen joints according to the type of disease course, at each time-point.

**FIGURE 7:**
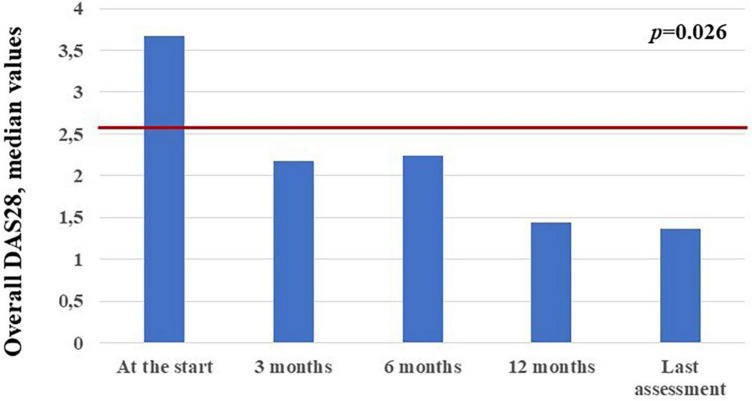
Median DAS28 values observed in the 14 patients with arthritis at the different time points. The red horizontal line indicates the DAS28 threshold below which arthritis may be considered in remission.

The mean DAS28 among patients with active arthritis at the start of canakinumab was 3.65 ± 1.15 at the start of canakinumab, 2.09 ± 0.91 after 3 months, 2.14 ± 1.14 at the 6-month visit, 1.4 ± 0.3 at the 12-month visit, 1.33 ± 0.75 at the last assessment (*p* = 0.026). No statistical differences were observed in the decrease of DAS28 at the 3-month visit, the 12 month-visit and at the last assessment according to the different disease patterns (*p* = 0.54, *p* = 0.77, *p* = 0.98, respectively). Figure 6C shows the mean DAS28 in the two study groups in different timepoints; Figure 7 highlights the overall decrease of the DAS28 in the whole group of patients.

The median PhGA was 40/100 (IQR = 60.5/100) at the start of treatment, 0/100 (IQR = 12.5/100) after 3 months, 0.5/100 (IQR = 11.25/100) at the 6-month assessment, 0/100 (IQR = 0/100) at the 12-month assessment, and 0/100 (IQR = 0/100) at the last assessment. The decrease of PhGA was statistically significant (*p* < 0.00001). No differences were observed in the decrease of PhGA between the systemic and the chronic-articular types at 3-month, 12-month, and last assessments (*p* = 0.48, *p* = 0.50, *p* = 0.69, respectively).

The PGA was 40/100 (IQR = 50/100) at the start of canakinumab, 10/100 (IQR = 19.5/100) after 3 months, 8.5/100 (IQR = 16.25/100) at the 6-month visit; 1/100 (IQR = 10/100) at the 12-month visit, and 0/100 (IQR = 10/100) at the last assessment. The decrease in the PGA was statistically significant (*p* = 0.004). No differences were observed in the decrease of PGA between the systemic and the chronic-articular types at 3-month, 12-month, and last assessments (*p* = 0.18, *p* = 0.95, *p* = 0.98, respectively).

### Glucocorticoid and cDMARDs sparing effect

The frequency of patients administered glucocorticoids was 20/26 at the start of treatment, 15/26 after 3 months, 7/24 at 12-month visit and 6/26 at the last assessment (*p* = 0.0002). No statistically significant differences were observed at the 3-month, 6-month, and at the last-assessment according to the different disease patterns (*p* = 0.68, *p* = 1.0, and *p* = 1.0, respectively).

**FIGURE 8:**
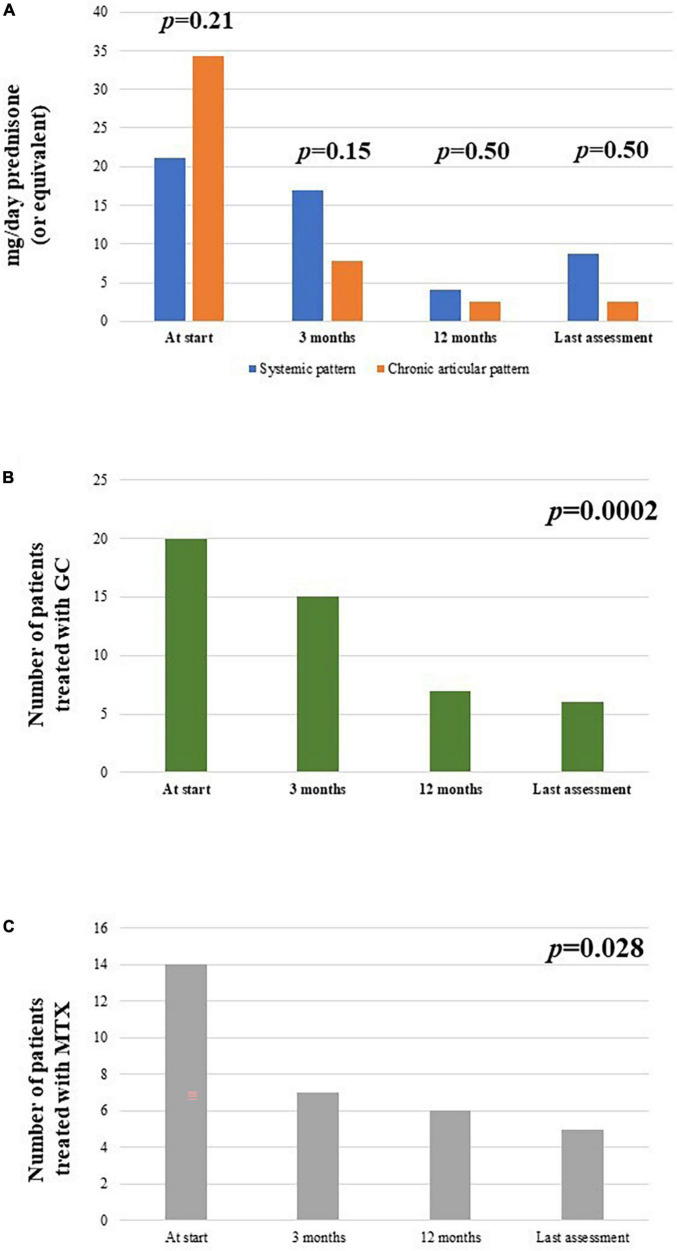
Description of **(A)** the median glucocorticoid (prednisone or equivalent) intake as mg/day at the start of canakinumab and at each follow-up visit, distinguishing according to the different disease pattern (systemic versus chronic-articular); **(B)** the total number of patients administered glucocorticoids (GC) at the different timepoints; **(C)** the total number of patients administered methotrexate (MTX) at the different timepoints.

The median glucocorticoids dosage (prednisone or equivalent) was 25 (IQR = 42) mg/day at the start, 5 (IQR = 7.5) mg/day after 3 months, 5 (IQR = 2.5) mg/day at the 6-month assessment, 2.5 (IQR = 2.5) mg/day at the 12-month visit and 2.5 (IQR = 2.5) mg/day at the last assessment (*p* < 0.00004). The overall reduction in glucocorticoid dosage from the start of canakinumab to the last follow-up visit was 93%. No differences were observed in the decrease of glucocorticoid dosage according with the different disease patterns at the 3-month assessment (*p* = 0.15, *p* = 0.50, and *p* = 0.50, respectively). Figure 8A represents the decrease in the number of patients requiring glucocorticoids at the different timepoints of the study and Figure 8B describes the daily glucocorticoids administered in patients already needing combination with steroids.

The cDMARDs were initially used with canakinumab in 14/26 patients; one patient started canakinumab together with methotrexate due to high disease activity. The number of patients administered cDMARDs decreased to 7/26 at 3-months and 6-month assessments, 6/24 at 12-month visit, 5/26 at the last follow-up; the number of patients treated with cDMARDs was significantly reduced at the last assessment if compared to the start of the treatment (*p* = 0.044). No statistically significant differences were observed in the frequency of cDMARDs use according with the different disease patterns pattern (*p* = 0.10, *p* = 1.00, and *p* = 1.00, respectively).

The cumulative methotrexate dosage used in all patients enrolled was 102.5 mg/week at the start of canakinumab (median value: 15 mg/week), 65 mg/week at the 3-month visit (median value: 15 mg/week), 60 mg/week at the 6-month visit (median value: 11.25 mg/week), 35 mg/week at the 12-month visit (median value: 12.5 mg/week), and 30 mg/week at the last assessment (median value: 7.5 mg/week). The overall reduction in methotrexate dosage from the start of canakinumab to the last follow-up visit was statistically significant (*p* = 0.023) and corresponded to 70.7%.

Figure 8C provides the number of patients administered methotrexate at the start of treatment and at the following visits.

### Changes in laboratory inflammatory markers

At the start of treatment, 24/26 patients showed an increase in the ferritin serum levels; 2 of them with systemic pattern presented a ferritin serum value higher than 3,000 mg/dl. At the 3-month assessment, all patients but one had normal serum ferritin levels; this patient showed normal levels at the 6-month assessment. At 6- and 12-month assessments and at the last follow-up visit none had abnormal ferritin serum levels.

The median serum ferritin value was 712 (IQR = 1705) ng/ml at the start of treatment, 95.2 (IQR = 181) ng/ml after 3 months, 75.6 (IQR = 99.75) ng/ml at the 6-month visit, 91.95 (IQR = 149) at the 12-month visit and 63 (IQR = 95) ng/ml at the last assessment. The decrease in the serum ferritin levels was statistically significant (*p* = 0.0002). No statistically significant differences were observed in the decrease of serum ferritin levels at the 3-month assessment between patients with systemic Still’s disease compared with patients with the chronic-articular type (*p* = 0.34).

The median ESR value was 50.5 (IQR = 25.75) mm/h at the start of canakinumab, 8 (IQR = 13) mm/h after 3 months, 6 (IQR = 12) mm/h at the 6-month visit, 5 (IQR = 7) mm/h at the 12-month visit, and 5 (IQR = 8) mm/h at the last assessment. The decrease of ESR values was statistically significant during the study period (*p* < 0.0001). No differences were observed in the decrease of ESR values at the 3-month assessment based on the disease pattern (*p* = 0.34).

The median CRP value was 7.16 (IQR = 64.2) mg/dl at the start of canakinumab, 0.28 (IQR = 2.57) mg/dl after 3 months, 0.43 (IQR = 1.4) mg/dl at the 6-month assessment, 0.4 (IQR = 0.85) mg/dl at the 12-month assessment, and 0.36 (IQR = 0.81) mg/dl at the last follow-up visit. The decrease in CRP values was statistically significant (*p* < 0.00001). No differences were observed in the decrease of CRP values at the 3-month assessment according to the different disease patterns (*p* = 0.48).

Inflammatory markers normalized in all but three patients at the 3-month assessment; no statistical differences were observed in the persistence of increased inflammatory markers between the systemic and the chronic-articular forms of the disease (*p* = 0.99).

**FIGURE 9:**
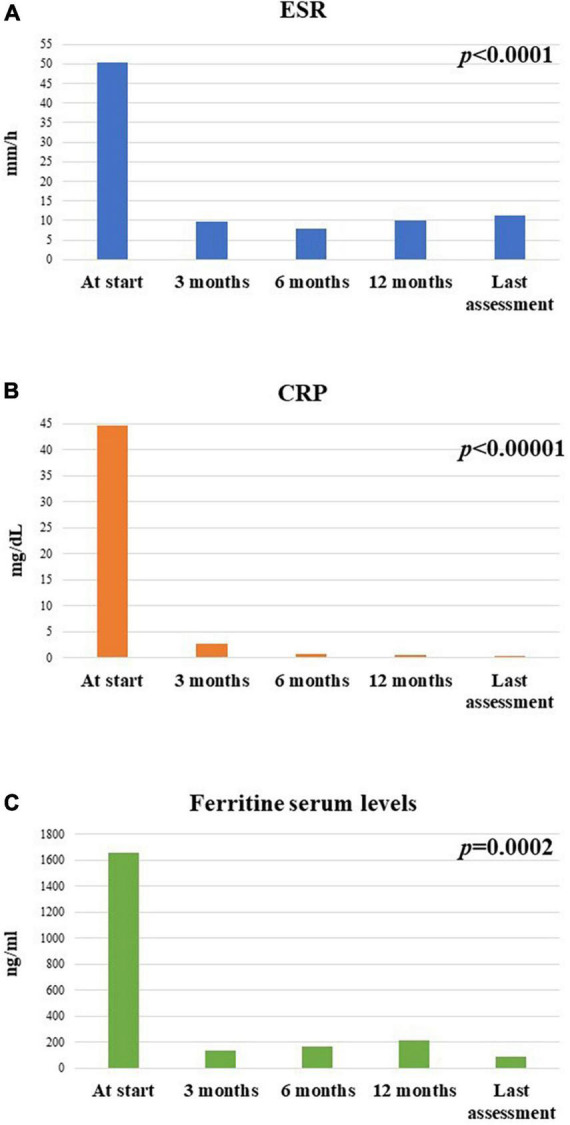
Mean **(A)** erythrocyte sedimentation rate (ESR), **(B)** C-reactive protein (CRP), and **(C)** ferritin serum levels at the different timepoints in the group of 26 patients enrolled in the study.

Figure 9 illustrates the median ESR, CRP and ferritin serum values collected in the whole cohort of patients at the different time-points.

Regarding other laboratory markers, an increase in transaminases was observed in 10 patients (8 with the systemic pattern) at the start of canakinumab; no patients had abnormal liver function enzymes at the following timepoints. Leukocytosis higher than 15,000 white blood cells/mm^3^ was observed in 11 patients (9 with the systemic pattern) at the start of canakinumab and in no patients at the following timepoints.

### Safety profile

The following four adverse events were reported during the follow-up period: episodes of dizziness and giddiness in one patient; occurrence of external otitis in one patient; heartburn associated with gastroesophageal regurgitation in one patient; a relapse of concomitant ulcerative colitis in one patient.

## Discussion

Inhibition of IL-1 or IL-6 has clearly demonstrated to be effective in controlling clinical and laboratory inflammatory manifestations of Still’s disease, with a strong glucocorticoid sparing effect and a tolerable safety profile (21, 24, 25). Despite the lack of internationally shared treatment guidelines, the current approach supports the use of IL-1 inhibitors in patients with Still’s disease (14, 16), while IL-6 antagonists may represent an effective treatment choice in refractory cases with persistent inflammatory joint involvement (15, 17). Different cytokine imbalances identified based on the different patterns of the disease (systemic versus chronic-articular) could suggest different molecular targets when establishing a personalized treatment approach in Still’s disease (26, 27). Looking at literature, this could partially explain why the frequency of non-responders to IL-1 inhibition has been found higher among patients with chronic-articular Still’s disease. Also, the systemic form of the disease and the absence of arthritis (or a lower number of arthritic joints) have been associated with a substantial response to IL-1 inhibition; conversely, the chronic-articular form and the presence of arthritis have been associated to a substantial response to IL-6 inhibition (15). Despite these premises, in our study the frequency of complete response to canakinumab was remarkably high disregarding the disease course. In particular, no statistically significant differences were observed when considering the overall frequency of complete response (with full control of clinical and laboratory manifestations), the retention in canakinumab therapy, the decrease of articular disease, and the glucocorticoid and cDMARD sparing effects. In the same way, the systemic Pouchot score, reflecting the typical Still’s disease manifestations, decreased in a significant way in the overall group and disregarding the type of Still’s disease.

Noteworthy, despite IL-1 inhibition has been supposed to be less suitable in patients with a prominent joint involvement (18, 19, 28, 29), articular items have proved to be responsive in the overall group, disregarding the type of disease course. Looking at literature, polyarticular involvement was observed to be a negative predictor for clinical response to IL-1 inhibition, especially when considering the persistence of arthritis (19, 28). Actually, the presence of polyarthritis could be associated to a loss of systemic inflammatory activity in favor of an autoimmune phenotype (29). In our cohort of patients, polyarticular forms are equally distributed in the two study subgroups. Therefore, the present study adds to previous experiences supporting the efficacy of canakinumab on DAS28 decrease (20, 30) and also highlights the absence of substantial differences between the systemic form and the chronic-articular form in the response of joint inflammation. In particular, DAS28 values, the number of tender and swollen joints, the PhGA and PGA proved to be overlapping in the two groups during the whole study period. However, we also point out the slower response of joint items in patients with the chronic-articular course: as observed in Figure 6, the number of arthritic joints and DAS28 values were significantly higher in the chronic-articular group at the 3-month assessment. Similarly, articular disease remission (DAS28 < 2.6) was reached by the systemic group as soon as the 3-month assessment, while the chronic-articular group reached disease remission starting from the 6-month visit. Despite this slower effect in the chronic-articular group, the control on joint inflammatory manifestations resulted to be overlapping with those observed in the systemic group in the subsequent follow-up. At further support of this, no significant differences were observed in the decrease of DAS28 between groups, straightening the superimposable response of joint involvement disregarding disease pattern. This should induce physicians to wait for the 6-month assessment before suspending canakinumab due to the only persistence of joint inflammation in chronic-articular disease.

The remarkable results in both types of Still’s course could be explained by the early canakinumab introduction. In this regard, the concept of a “window of opportunity,” namely, as the period of time after the disease onset during which starting IL-1 inhibition may be more effective, has been proposed for patients with Still’s disease, at least for patients with pediatric onset (19, 31–33). Saccomanno et al. identified a disease duration ≤ 3.9 years as cut-off beneath which patients with sJIA were more likely completely responsive to the IL-1 receptor antagonist anakinra (33). Horneff et al. also observed that patients treated with IL-1 inhibitors within 12 months from disease onset achieved clinical remission more frequently than patients starting the treatment thereafter (34). A further experience assessing any window of opportunity in 141 AOSD patients treated with anakinra identified a good effectiveness disregarding the time between disease onset and the start of IL-1 inhibition. Nevertheless, a faster effectiveness of anakinra in articular manifestations was observed in patients undergoing an early IL-1 inhibition (22). These results flanked those provided by Cavalli et al. about the dramatic clinical improvement on arthritis in patients with Still’s disease undergoing canakinumab as a first-line treatment (35). Supported by these several evidences, we can speculate that the excellent results obtained with joint indexes in both systemic and chronic-articular still’s disease could be related to the early canakinumab administration. This topic should be addressed by future targeted studies.

Beyond the effectiveness on clinical manifestations, canakinumab allowed a complete control of laboratory inflammatory parameters irrespective of the type of disease course, thus confirming a previous similar finding (28). We evaluated this endpoint only between the time at canakinumab introduction and the 3-month assessment. Actually, laboratory inflammatory markers usually reduce dramatically in the first months of canakinumab treatment, remaining substantially reduced thereafter. For this reason, the evaluation of canakinumab effectiveness on laboratory features between groups was more sensible and reasonable during this time.

The excellent results on clinical and laboratory manifestations were obtained despite the significant sparing of glucocorticoids both in terms of patients requiring daily steroids and in terms of daily dosage among patients still needing this concomitant treatment. In the same way a slow but steady reduction was observed in the frequency of use and in the weekly methotrexate dosage. This is a central point highlighting the possibility to decrease the immunosuppressant load in patients with Still’s disease, which is essential to reduce adverse events, previously described more frequent in patients administered with IL-1 antagonists and a concomitant cDMARD (36).

We have meshed together adult-onset and pediatric Still’s disease, as they have been identified as the same disease arising in different ages (2, 3). At support of this, a recent Bayesian and population model-based analysis has pointed out a similarity of clinical outcomes in patients with sJIA and AOSD treated with canakinumab (37). Other clinical experiences have also supported the concept of a continuum of Still’s disease irrespective of the age at disease onset, with no differences in canakinumab response between sJIA and AOSD regarding the frequency of complete response and the relapse rates (38, 39). On this basis, we also performed a unique data analysis disregarding the age at disease onset.

The limits of this study consist of those that typically affect retrospective studies. Despite being drawn from an international registry (23), the number of patients involved in this study remains not particularly large. This is related to the rarity of Still’s disease and the reduced propensity to use canakinumab as first-line biotechnological agent because of healthcare spending issues. Nevertheless, this is a real-world study performed on a small slice of hard-to-enroll patients to address an already unmet need for everyday clinical practice.

In conclusion, canakinumab used for Still’s disease has proved to be effective in controlling both clinical and laboratory manifestations disregarding the type of disease course when used as first-line biotechnological agent. Canakinumab could show a slower efficacy on joint manifestations in chronic-articular Still’s disease; however, articular disease control is equally obtained in both groups in the long term and the initial persistence of isolated arthritis should not induce the treatment withdrawal in chronic-articular patients. These excellent results might have been further enhanced by the early start of IL-1 inhibition and should draw attention to the concept of window of opportunity, especially in chronic-articular Still’s disease. Targeted studies should be conducted in the near future to better clarify this concept.

## Data availability statement

The raw data supporting the conclusions of this article will be made available by the authors, without undue reservation.

## Ethics statement

The study has been approved by the Ethics Committee of Azienda Ospedaliero Universitaria Senese, Siena, Italy (AIDA Project; Ref. N. 14951) as part of the AIDA Program. Written informed consent/assent was obtained from all patients at the time of the recruitment into the AIDA Registry for Still’s disease. Written informed consent from the patients or legal guardian/next of kin was obtained to participate in this study, in accordance with the National and European legislation and the institutional requirements.

## Author contributions

AV and VC wrote the first draft of the manuscript and conceived and designed the study. AV, VC, MM, GL, GE, JS, FL, PR, EB, GC, IM, CG, FC, LD, CC, RG, AB, KL, BF, FI, and LC were included based on the number of AOSD patients treated with canakinumab recruited in the AIDA Registries by 1st September 2022. CF, AT, GR, IA, PS, EW-S, and RP were included in the authorship as investigators from the top contributor centers for any of the other eight AIDA Registries. AB was the bioengineer involved in the technical management of the platform and registries. LC took care of the final revision of the manuscript and accounted for AIDA Registries Coordinator. All authors contributed to the article and approved the submitted version.
